# The novel circRNA circ_0045881 inhibits cell proliferation and invasion by targeting mir-214-3p in triple-negative breast cancer

**DOI:** 10.1186/s12885-024-12007-0

**Published:** 2024-03-01

**Authors:** Jie Ren, Wei Chen, Ya Zhou, Jianxiong Sun, Guoqin Jiang

**Affiliations:** 1 Department of General Surgery, The Second Affiliated Hospital of Soochow University, No. 1055 Sanxiang Road, 215004 Suzhou, Jiangsu Province China; 2 Surgery Department, Suzhou Wuzhong People’s Hospital, 215128 Suzhou, Jiangsu Province China

**Keywords:** Triple-negative breast cancer, circRNA, hsa_circ_0045881, miR-214-3p, Invasion, Migration

## Abstract

**Background:**

Triple-negative breast cancer (TNBC) is the most lethal subtype of breast cancer (BC). The circRNA-miRNA‒mRNA axis is a promising biomarker for the early diagnosis and prognosis of BC. However, the critical circRNA mediators involved in TNBC progression and the underlying regulatory mechanism involved remain largely unclear.

**Methods:**

In this study, we carried out a circRNA microarray analysis of 6 TNBC patients and performed a gene ontology (GO) analysis. Kyoto Encyclopedia of Genes and Genomes (KEGG) analysis was used to characterize important circRNAs involved in TNBC progression. The interaction between circRNAs and miRNAs was determined by dual luciferase and RNA immunoprecipitation (RIP) assays. Moreover, Transwell, wound healing and Cell Counting Kit-8 (CCK8) assays were performed with altered circRNA or miRNA expression in MDA-MB-231 and BT-549 cells to investigate the roles of these genes in cell invasion, migration and proliferation.

**Results:**

A total of 78 circRNAs were differentially expressed in TNBC tissues, and the hsa_circ_0045881 level was significantly decreased in TNBC tissues and cells. Lentivirus-mediated hsa_circ_0045881 overexpression in MDA-MB-231 and BT-549 cells significantly reduced cell invasion and migration capacity. Additionally, hsa_circ_0045881 interacted with miR-214-3p in MDA-MB-231 cells. miR-214-3p mimics in MDA-MB-231 and BT-549 cells significantly enhanced cell invasion, migration and proliferation, but the other combinations of inhibitors had opposite effects on cell activity.

**Conclusions:**

Our data indicated that the circRNA has_circ_0045881 plays key roles in TNBC progression and that hsa_circ_0045881 might act as a sponge for miR-214-3p to modulate its levels in TNBC cells, thereby regulating cell invasion, metastasis and proliferation. hsa_circ_004588 might be a potential prognostic marker and therapeutic target for TNBC.

**Supplementary Information:**

The online version contains supplementary material available at 10.1186/s12885-024-12007-0.

## Background

BC is the most prevalent cancer in females worldwide and poses a major threat to women’s health. More than 1 million women worldwide are diagnosed with BC, and more than 500 thousand people die annually [1, 2]. TNBC is a type of BC that is associated with high-risk clinical manifestations, such as early haematogenous metastasis, aggressive behaviour and a high incidence of visceral cancer [3]. TNBC tumour cells are prone to drug resistance, which leads to poor clinical treatment efficacy [4]. Therefore, clarifying whether there are specific regulators involved in the occurrence and development of TNBC, how TNBC plays a highly invasive pathological role, and how to identify molecular targets that can be used for effective clinical treatment of TNBC are important directions for basic research and precision treatment of TNBC.

There are many noncoding RNAs (ncRNAs) in the human genome that play important roles in the occurrence and development of many diseases. Circular RNAs (circRNAs) are newly recognized ncRNAs without 5’ caps or 3’ poly (A) tails [5]. They form a closed continuous loop by covalent bonds and were first found in eukaryotic cells as early as the 1970s [6]. Due to detection limitations, circRNAs did not receive enough attention during this period. In recent years, with the development and application of bioinformatics and RNA sequencing technology, circRNAs have been found to be abundant and closely related to the occurrence and development of a variety of human diseases [7]. Accumulating evidence suggests that circRNAs act as microRNA (miRNA) sponges by competitively binding to miRNAs and preventing the silencing of target genes [8, 9]. For instance, hsa_circ-ERBIN was shown to aggravate colorectal cancer by sponging miR-125a-5p and miR-138-5p and alleviating translation initiation Factor 4E binding protein 1 (4EBP-1) silencing [10]. Moreover, hsa_circ_001783 was shown to promote the development of BC by sponging miR-200c-3p [11]. To further explore the mechanism underlying the occurrence and progression of TNBC, an increasing number of researchers are investigating circRNAs, which are expected to be novel markers and therapeutic targets for TNBC.

The circRNA/miRNA/mRNA axis has attracted increased amounts of attention because it may be a molecular marker for the early diagnosis and prognosis of BC [12]. miRNAs are a class of ∼21–22 nucleotide (nt) small ncRNAs that stimulate the degradation of mRNAs, regulate gene expression and inhibit translation by interacting with the 3′ untranslated regions (UTRs) of targets, and function in a series of essential biological processes [13–17]. miR-214 is transcribed by Dynamin 3, and previous research has demonstrated that miR-214-3p is closely associated with cancer, adipogenesis, skeletal muscle development, etc. [18].. Increased expression of miR-214-3p reduces proliferation, invasion and migration in hepatocellular carcinoma [15, 19]. Furthermore, miR-214-3p overexpression significantly downregulated bone morphogenetic protein 2 (BMP2) expression and osteoblast proliferative activity [20]. Recently, it was reported that the osteoclastic miR-214-3p level was upregulated in BC patients, which subsequently promoted the proliferation of BC cells [21, 22]. Moreover, miR-214-3p was abnormally enhanced in TNBC cells, and a low level of miR-214-3p was found to inhibit the invasion and migration of TNBC cells [23]. Although several circRNAs have been shown to participate in the pathogenesis of BC, novel circRNA mediators and the regulatory mechanism underlying circRNA/miR-214-3p interaction still need further investigation to advance the understanding of the pathogenesis of TNBC.

In this study, we conducted circRNA expression profile analysis in TNBC patients and found that hsa_circ_0045881 was dramatically decreased. Lentivirus-mediated hsa_circ_0045881 overexpression in MDA-MB-231 and BT-549 cells significantly reduced invasion and migration capacity. Moreover, we further confirmed that hsa_circ_0045881 interacted with miR-214-3p in TNBC cells. MDA-MB-231 and BT-549 cells transiently transfected with the miR-214-3p mimic promoted cell invasion, migration and proliferation, but the miR-214-3p inhibitor exerted the opposite effects. Therefore, our data indicate that hsa_circ_0045881 is closely associated with human TNBC progression and that the hsa_circ_0045881/miR-214-3p axis plays vital roles in invasion pathology. Thus, hsa_circ_0045881 might serve as a novel prognostic and therapeutic target for TNBC treatment in the future.

## Methods

### Tissues and cell lines

Twenty-nine pairs of fresh TNBC tissues and adjacent noncancerous tissues were obtained from patients during surgery at the Second Affiliated Hospital of Soochow University (Suzhou, China) from January 2014 to July 2019. This study was approved by the Ethics Committee of the Second Affiliated Hospital of Suzhou University. All TNBC patients were diagnosed pathologically by intraoperative or postoperative pathology. None of the patients had been administered chemoradiotherapy or radiotherapy before the surgery. Intraoperative TNBC and adjacent tissues were placed in enzyme-free EP tubes containing RNA fixative and stored at -80 °C. Six pairs of tissue samples were subjected to circRNA microarray analysis, and the other 23 pairs were subjected to further investigation in TNBC.

The human TNBC cell lines BT-549, MCF-10 A, MDA-MB-231 and MDA-MB-468 were acquired from American Type Culture Collection (ATCC, USA) and were cultured in Dulbecco’s modified Eagle’s medium (DMEM) (Gibco, Carlsbad, CA, USA) supplemented with 10% foetal bovine serum (FBS; HyClone, Invitrogen) and 100 U/ml penicillin in a humidified incubator at 37 °C with 5% CO_2_.

### Expression profile analysis of circRNAs

Total RNA from each sample was quantified using a NanoDrop ND-1000. Sample preparation and microarray hybridization were performed based on Arraystar’s standard protocols. Total RNA was digested with RNase R (Epicentre, Inc.) to remove linear RNAs and enrich circular RNAs. The enriched circular RNAs were subsequently amplified and transcribed into fluorescent cRNAs utilizing a random priming method (Arraystar Super RNA Labelling Kit, Arraystar). The labelled cRNAs were hybridized onto the Arraystar Human circRNA Array V2 (8 × 15 K, Arraystar). After the slides were washed, the arrays were scanned with an Agilent G2505C scanner.

Agilent Feature Extraction software (version 11.0.1.1) was used to analyse the acquired array images. Quantile normalization and subsequent data processing were performed using the R software package limma. Significantly differentially expressed circRNAs between the two groups were identified through volcano plot filtering. Differentially expressed circRNAs between two samples were identified through fold change filtering. Hierarchical clustering was performed to visualize the distinguishable circRNA expression patterns among the samples.

Microarray procedures and analysis were performed by KangChen Biotech (Shanghai, China).

### Quantitative real-time PCR (qRT‒PCR)

Total RNA was isolated from cells and tissues using TRIzol (Takara) following the manufacturer’s protocol. The primers used are shown in Table 1. Total RNA (1 µg) was used to synthesize cDNA with a PrimeScript II 1st Strand cDNA Synthesis Kit (Takara, Beijing, China). Gene expression levels were quantified by the 2^−ΔΔCT^ method, with GAPDH serving as an internal reference.

**Table 1:** Sequence information for primers used in qRT-PCR

Gene	Forwad primer(5’-3’)	Reverse primer(5’-3’)
hsa_circ_0001020	GAGCAACACCTGGACTTCAAA	TCCATGTGACCATCTCCTGT
hsa_circ_0058741	CACTGTGGAAGGGAAGGAAG	AGGCTCTGTGGCTGTAGGC
hsa_circ_405836	GTATGTTTAGTTCCAATCGTCAG	ACATTATGGGTACTGAAGCAA
hsa_circ_0000148	AGACCTATCCACCACTCCCA	CCTGCCATTTCCTCCTCC
hsa_circ_0005019	ATCTGAAGGCGCTCACGCACT	AGATCCGGCCACCTGAACACTT
hsa_circ_0027089	GGGTGGTGATGAGGATGTAGA	TCATCTTCCCAGTCTTTCCAATT
hsa_circ_0035445	CCCACAGTGAGTCAGGTTGA	AAGGAGATCACTTTGGCCAA
hsa_circ_0045881	CCTTGCCTTTCTTCCTGTTC	CCCTTCGCCGTCTCCAT
ACTB	CGTGGACATCCGCAAAGA	GAAGGTGGACAGCGAGGC

### Plasmid construction, lentivirus packaging and cell transfection

To construct the hsa_circ_0045881 overexpression vector, full-length human circ_0045881 was inserted into the pcDNA3.1(+) vector (GenePharma Co., Ltd., China), which contains a front and back circular frame, whereas the mock vector was used as a control. For lentivirus packaging, the hsa_circ_0045881 overexpression vector was constructed, and the lentivirus shuttle plasmid and carrier plasmid were prepared. The packaged lentivirus (GenePharma Co., Ltd., China) was subsequently used to infect MDA-MB-231 and BT-549 cells at an MOI of 10.

### Transwell assay

MDA-MB-231 and BT-549 cells transfected with OE-circ_0045881, the miR-214-3p mimic or inhibitor and the corresponding controls (2 × 10^4^) were plated in the upper chamber of a transwell plate. RPMI-1640 medium containing FBS (10%) was added to the lower chamber. Cells in the lower chamber were imaged and counted after fixation with formaldehyde (4%) and staining with crystal violet (0.1%). Three additional fields of view were observed for counting.

### Wound healing assay

Compared with the corresponding normal controls, MDA-MB-231 and BT-549 cells were transfected with OE-circ_0045881, the miR-214-3p mimic or the inhibitor and seeded. Then, a linear wound was scratched on the cell monolayer with a sterile pipette tip. Twenty-four hours later, the migrating cells were imaged, and the wound width was statistically analysed to quantify the migrating capacity.

### Cell counting kit-8 (CCK8) assay

A CCK8 assay was also conducted following the manufacturer’s instructions (WST-8, Japan). MDA-MB-231 and BT-549 cells were transfected with OE-circ_0045881, the miR-214-3p mimic or the inhibitor, and the corresponding controls were incubated in a 96-well plate at a density of 3000 cells/well. A total of 10 µl of CCK8 reagent was added to each well at 24, 48, 72, and 96 h, after which the cells were incubated for 2 h. The absorbance at 450 nm was determined using a spectrophotometer.

### RIP assay

RIP was performed using a Magna RIP RNA-Binding Protein Immunoprecipitation Kit (Millipore, USA). MDA-MB-231 cell lysates (2 × 10^7^) were prepared with 50 µl of RIP lysis buffer and centrifuged at 2500 × g for 15 min. The supernatant was subsequently transferred to RNA-free 1.5 ml centrifuge tubes and incubated with 1 ml of RIP wash buffer. The procedures were performed according to the manufacturer’s instructions. miR-1178-3p was used as a negative control. The retrieved miR-214-3p levels absorbed by the magnetic beads were detected via qRT‒PCR and normalized to the input.

### Dual luciferase assay

The binding sites between hsa_circ_0045881 and miR-214-3p were predicted via CircInteractome (https://circinteractome.nia.nih.gov/). miR-214-3p was cloned and inserted into a dual-luciferase reporter vector. MDA-MB-231 cells were seeded in 48-well plates, kept in a humid incubator for 24 h and then cotransfected with luciferase reporter plasmids and Renilla plasmids with Lipofectamine 2000 (Invitrogen, USA). The firefly and Renilla luciferase activities were analysed with the Dual-LuciferaseTM Reporter Assay System (Promega, USA) 48 h after transfection, according to the manufacturer’s instructions.

### Statistical analysis

All the experiments were repeated at least three times in this study. The data were analysed using Student’s t test and ANOVA with SPSS 15.0 software (SPSS, Chicago, IL, USA). All the results are summarized and presented as the means ± standard deviations (SDs). A P value < 0.05 was considered to indicate statistical significance.

## Results

### Analysis of circRNA expression profiles

**Fig. 1:**
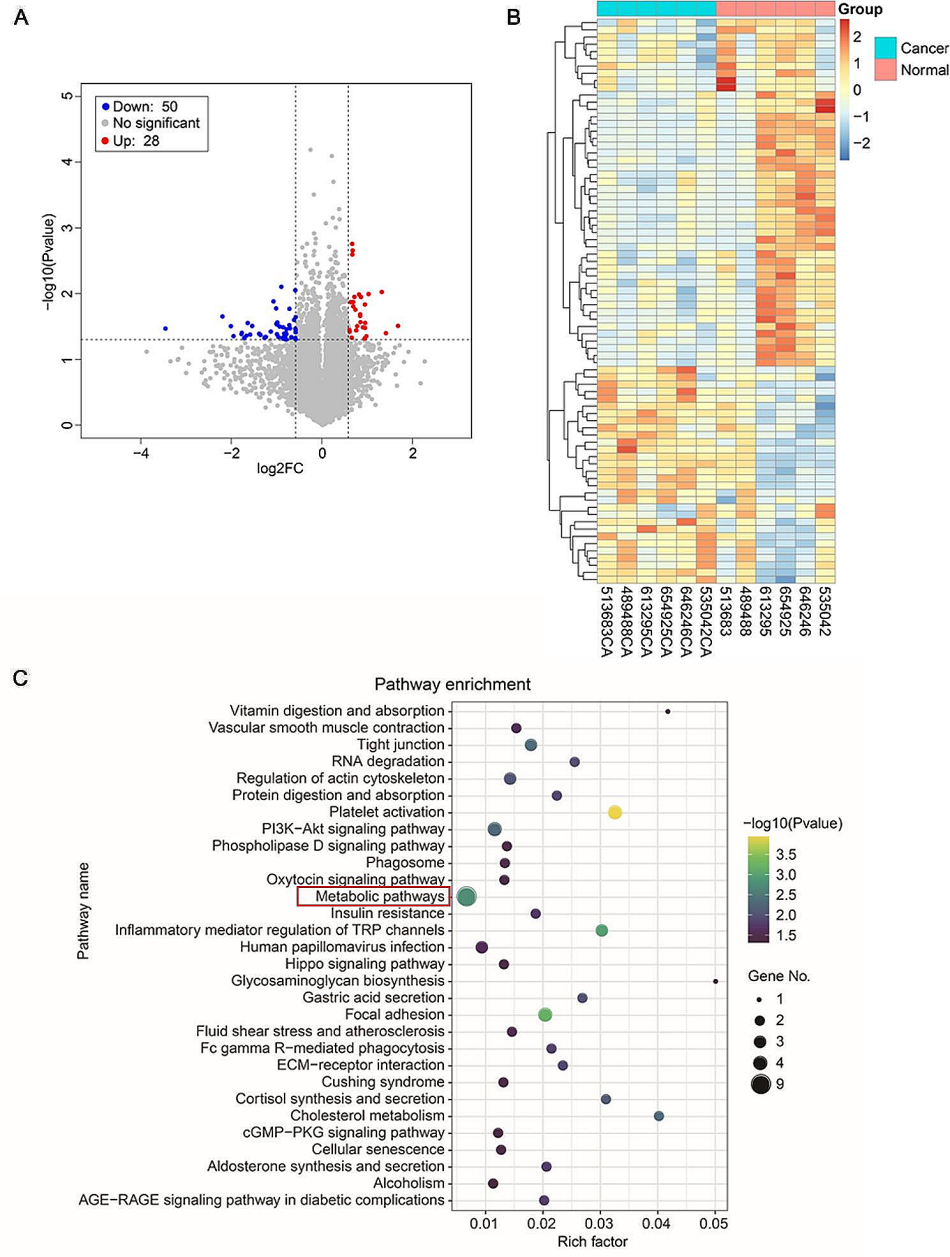
Analysis of circRNA expression profiles. (**A**) and (**B**) The volcano plot (**A**) and heatmap (**B**) show that 78 circRNAs were significantly expressed in TNBC patients. Red and blue represent upregulated and downregulated circRNAs, respectively. () KEGG analysis of the differentially expressed circRNAs **C** in TNBC patients. X-axis, Rich factor; Y-axis, the top 30 enriched KEGG pathways

To identify specific circRNAs that are differentially expressed between TNBC tissues (cancer) and adjacent noncancerous tissues (normal), 6 pairs of tissue samples pooled from TNBC patients were subjected to a circRNA microarray assay. A total of 78 circRNAs that were differentially expressed by > 1.5-fold in TNBC tissues compared to normal tissues were identified; 28 were upregulated, and 50 were downregulated, as shown in the volcano plot and heatmap (Fig. 1A, B). Furthermore, we carried out GO and KEGG analyses and detected 8 differentially expressed circRNAs enriched in metabolic pathways (Fig. 1C, Fig. S1).

**Fig. 2:**
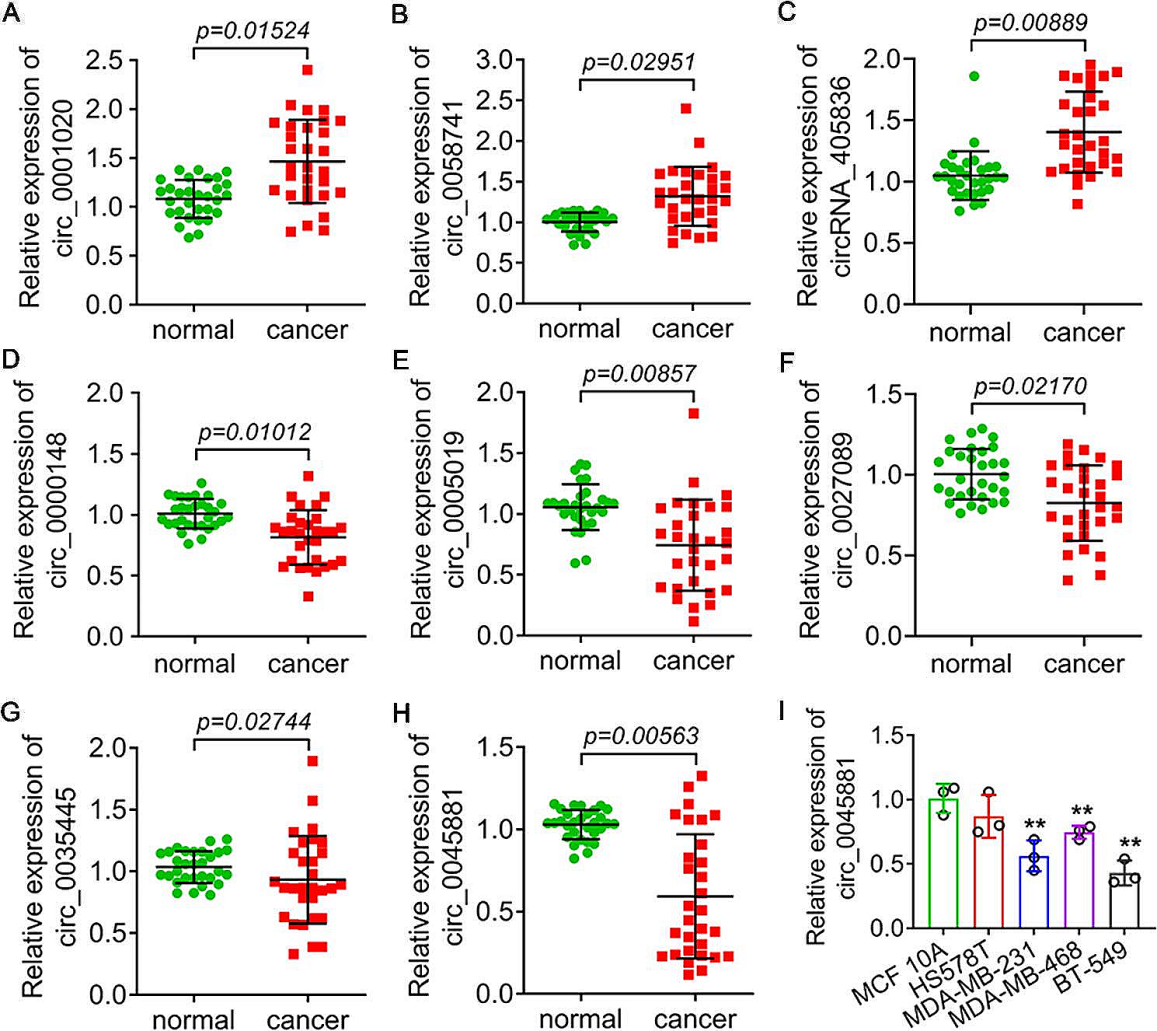
The expression of 8 circRNAs in TNBC patients and cells. (A-H) The relative expression of has_circ_0001020 (**A**), has_circ_0058741 (**B**), has_circ_405836 (**C**), has_circ_0000148 (**D**), has_circ_0005019 (**E**), has_circ_0027089 (**F**), has_circ_0035445 (**G**), and has_circ_0045881 (**I**) in 23 TNBC patients. (**I**) The relative expression of has_circ_0045881 in different TNBC cells. The data are presented as the means ± SDs; n ≥ *3*, **P < *0.01*

To confirm these findings, we detected the expression of these 8 circRNAs in TNBC tissues from 23 patients. The qRT‒PCR results revealed that 3 circRNAs, hsa_circ_0001020, hsa_circ_0058741, and hsa_circ_405836, were significantly upregulated, and 5 circRNAs, hsa_circ_0045881, hsa_circ_0035445, hsa_circ_0027089, hsa_circ_0005019 and hsa_circ_0000148, were significantly downregulated (*P* < *0.05*) (Fig. 2A‒H). Among these 8 circRNAs, hsa_circ_0045881 exhibited strongly reduced expression (*P* < *0.01*) (Fig. 2H). We subsequently narrowed the scope of the analysis to this gene and detected its expression in different TNBC cells. The results showed that the hsa_circ_0045881 expression level decreased significantly in MDA-MB-231, MDA-MB-468 and BT-549 cells compared to MCF-10 A cells (**P < *0.01*) (Fig. 2I). MDA-MB-231 and BT-549 cells were subjected to further study. Patients were grouped into low-hsa_circ_0045881 and high-hsa_circ_0045881 groups, with their average expression in tissues serving as the cut-off. Notably, a decreased expression of hsa_circ_0045881 was shown to be associated with a large tumour size (*p* = 0.043), advanced TNM stage (*p* = 0.015), high Ki-67 proportion (> 20%) (*p* = 0.002), increased lymphatic metastasis (*p* = 0.029) and death in individuals (*p* = 0.006) with TNBC (Table 2).

**Table 2:** Association of hsa_circ_0045881 expression with clinical features of TNBC patients

Items	High (*n* = 13)	Low (*n* = 16)	χ^2^	P
Age (years old)			0.14	0.712
< 50	5	7		
≥ 50	8	9		
Tumor size (cm)			5.70	0.043*
< 2	9	6		
≥ 2	4	10		
TNM stage			6.33	0.015*
I-II	8	5		
III	5	11		
Ki67			8.62	0.003**
< 20%	10	4		
≥ 20%	3	12		
Lymphatic metastasis			5.98	0.029*
N_0 − 1_	7	5		
N_2 − 3_	6	11		
Survival			8.04	0.006**
Yes	12	12		
No	1	4		

*P < *0.05*, **P < *0.01*

### Overexpression of hsa_circ_0045881 inhibited invasion and migration in MDA-MB-231 and BT-549 cells

**Fig. 3:**
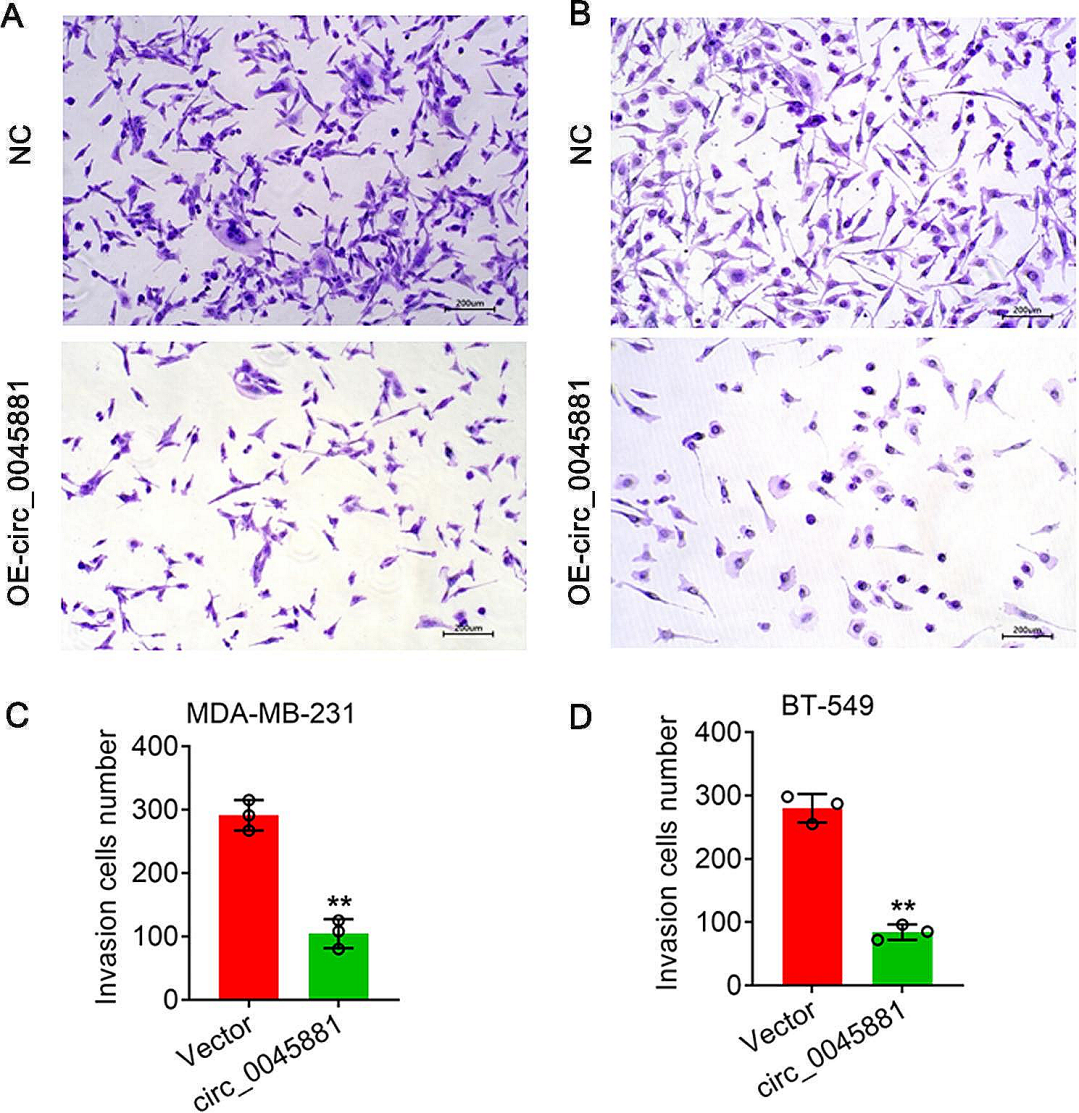
Detection of cell invasion by transwell experiments. (**A**) and (**B**) Invasion of MDA-MB-231 (**A**) and BT-549 (**B**) cells with lentivirus-induced circ_0045881 overexpression. (**C**) and (**D**) Quantitative analysis of cell invasion in A and B. Bars = 200 μm. The data are presented as the means ± SDs; n ≥ *3*, **P < *0.01*

**Fig. 4:**
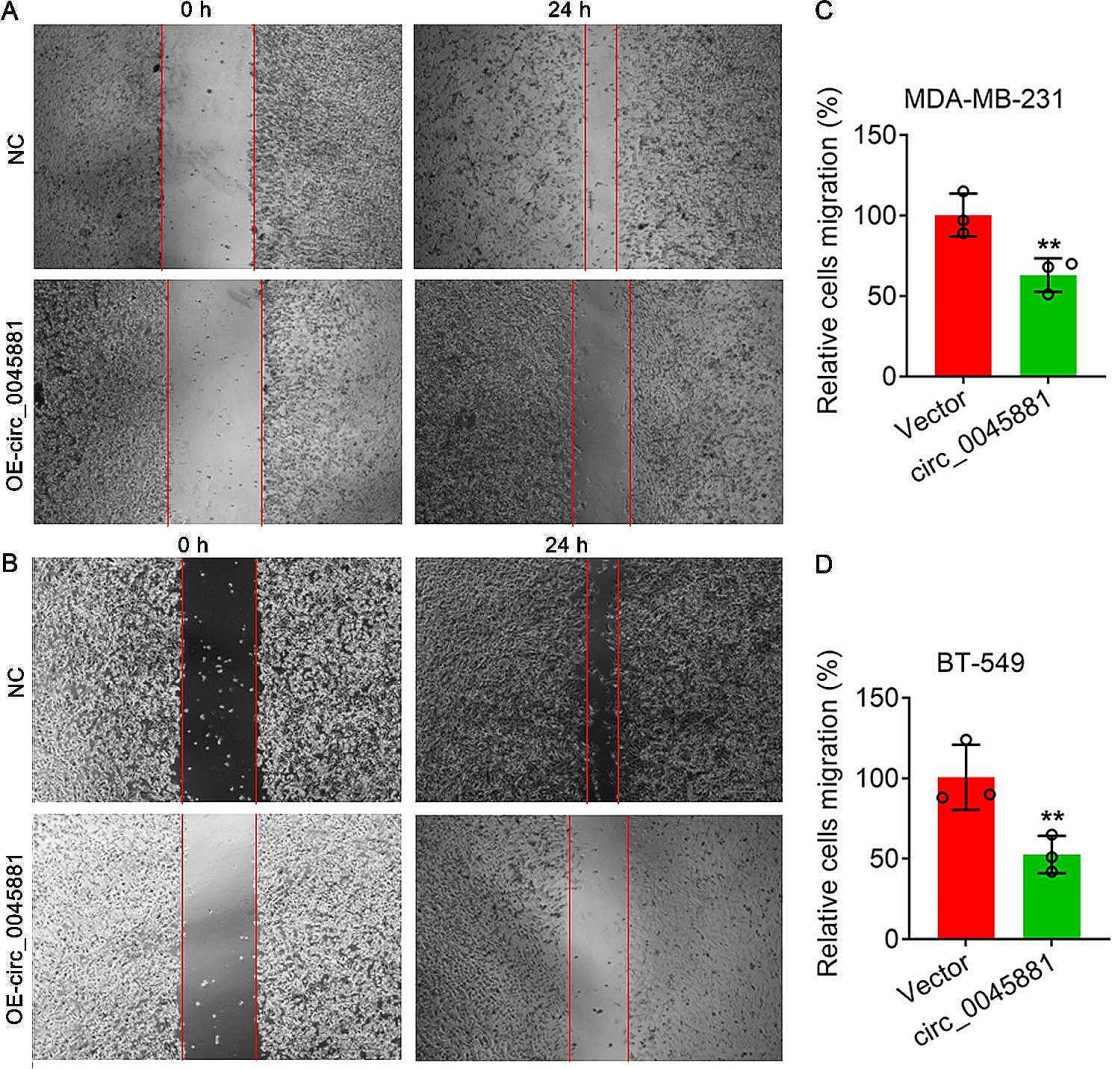
Detection of cell migration via a wound healing assay. (**A**) and () Migration of MDA-MB-231 (**A**) and BT-549 (**B**) cells with lentivirus **B** -induced has_circ_0045881 overexpression. (**C**) and (**D**) Quantitative analysis of cell migration in **A** and **B**. Scale bars = 200 μm. The data are presented as the means ± SDs; n ≥ *3*, **P < *0.01*

To determine the biological function of hsa_circ_0045881 in TNBC, we overexpressed hsa_circ_0045881 in MDA-MB-231 and BT-549 cells via lentivirus-mediated transduction (Fig. S2). The expression levels of hsa_circ_0045881 in MDA-MB-231 and BT-549 cells were significantly increased (**P < *0.01*) (Figs. S2A-D). Transwell assays showed that, compared with the normal controls, OE-circ_0045881 significantly reduced the invasion ability of both MDA-MB-231 and BT-549 cells (**P < *0.01*) (Fig. 3A-D). Additionally, the OE-circ_0045881 group exhibited obviously wider blank scratches after 24 h, which indicated that the migration ability of both the MDA-MB-231 and BT-549 cells overexpressing hsa_circ_0045881 was significantly decreased. (**P < *0.01*) (Fig. 4A-D).

### The interaction between hsa_circ_0045881 and miR-214-3p

**Fig. 5:**
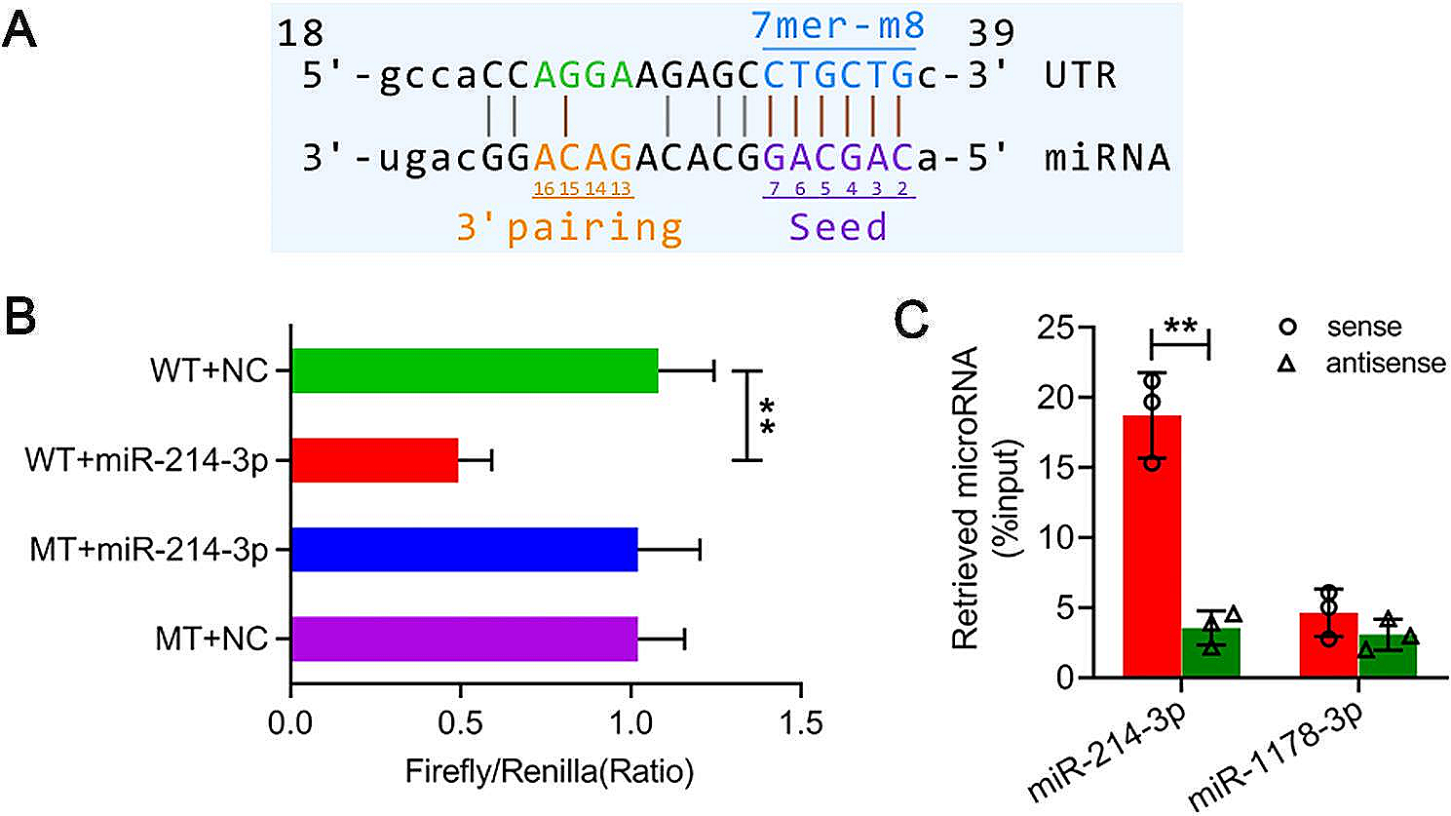
The interaction between has_circ_0045881 and miR-214-3p in TNBC cells. (**A**) Diagram showing the binding site between hsa_circ_0045881 and miR-214-3p. (**B**) The firefly and Renilla luciferase assays showed that the binding of has_circ_0045881 and miR-214-3p was controlled by MT and NC in MDA-MB-231 cells. MT, has_circ_0045881 mutation. NC, samples treated without miR-214-3p. (**C**) The RIP results showed that the immunoprecipitation of miR-214-3p by has_circ_0045881 (sense) was controlled by has_circ_0045881 (antisense) in MDA-MB-231 cells. miR-1178-3p was used as a negative control. The data are presented as the means ± SDs; n ≥ *3*, **P < *0.01*

Considering that hsa_circ_0045881 and miR-214-3p play important roles in TNBC development, we carried out dual luciferase assays and RIP analysis to investigate the interaction between these two ncRNAs (Fig. 5). The binding sites between hsa_circ_0045881 and miR-214-3p were predicted according to bioinformatics analysis (CircInteractome, https://circinteractome.nia.nih.gov/) and are shown in Fig. 5A. The firefly and Renilla dual luciferase activities were significantly lower in the WT + miR-214-3p groups than in the WT + NC group (**P < *0.01*) (Fig. 5B); MT + miR-214-3p and MT + NC were used as controls (Fig. 5B). Furthermore, the enrichment of miR-214-3p compared to that of the input was significantly greater when miR-214-3p was immunoprecipitated with the sense probe hsa_circ_0045881 than when it was immunoprecipitated with antisense probes (**P < *0.01*) (Fig. 5C). Additionally, hsa_circ_0045881 expression did not significantly change in response to the miR-214-3p inhibitor in MDA-MB-231 or BT-549 cells (Fig. S3).

### Mir-214-3p promoted invasion and migration in MDA-MB-231 and BT-549 cells

To clarify the role of miR-214-3p in regulating cell activity, we transfected miR-214-3p mimics and inhibitors into MDA-MB-231 and BT-549 cells, respectively. The expression of miR-214-3p was measured by qRT‒PCR, and miR-214-3p was downregulated significantly in MDA-MB-231 cells transfected with inhibitor-1 and − 2 (**P < *0.01*) (Fig. S4A). Considering that inhibitor-1 reduced miR-214-3p expression to relatively lower levels, we used this inhibitor in the following study. Additionally, miR-214-3p expression was significantly promoted in MDA-MB-231 cells after mimic transfection (**P < *0.01*) (Fig. S4B).

**Fig. 6:**
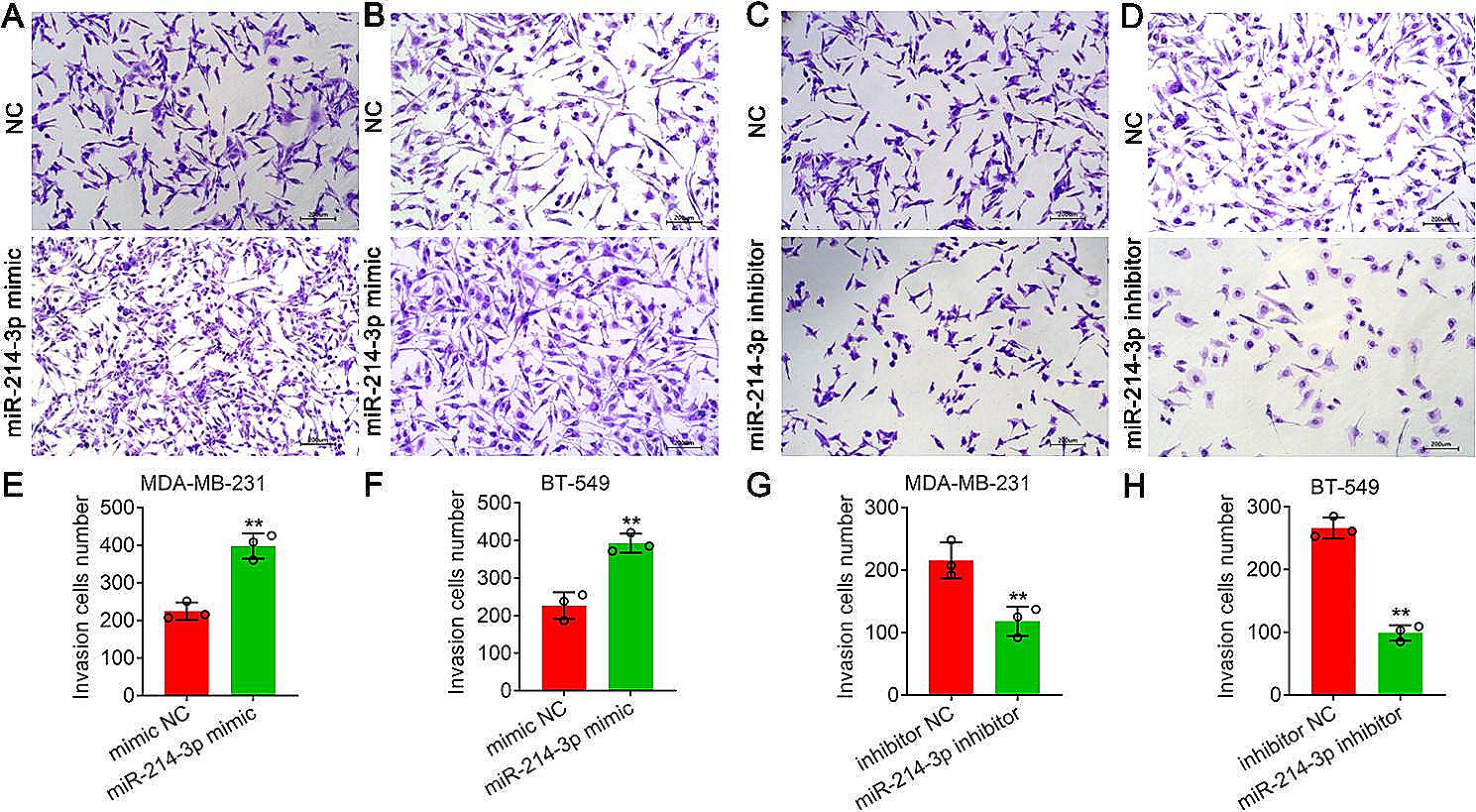
Detection of cell invasion by transwell experiments. (**A**) and (**B**) Invasion of MDA-MB-231 (**A**) and BT-549 (**B**) cells transiently transfected with the miR-214-3p mimic. (**C**) and (**D**) Invasion of MDA-MB-231 (**C**) and BT-549 (**D**) cells transiently transfected with miR-214-3p inhibitors. (**E**) and (**H**) Quantitative analysis of cell invasion in A, B, C and D. Bars = 200 μm. The data are presented as the means ± SDs; n ≥ *3*, **P < *0.01*

**Fig. 7:**
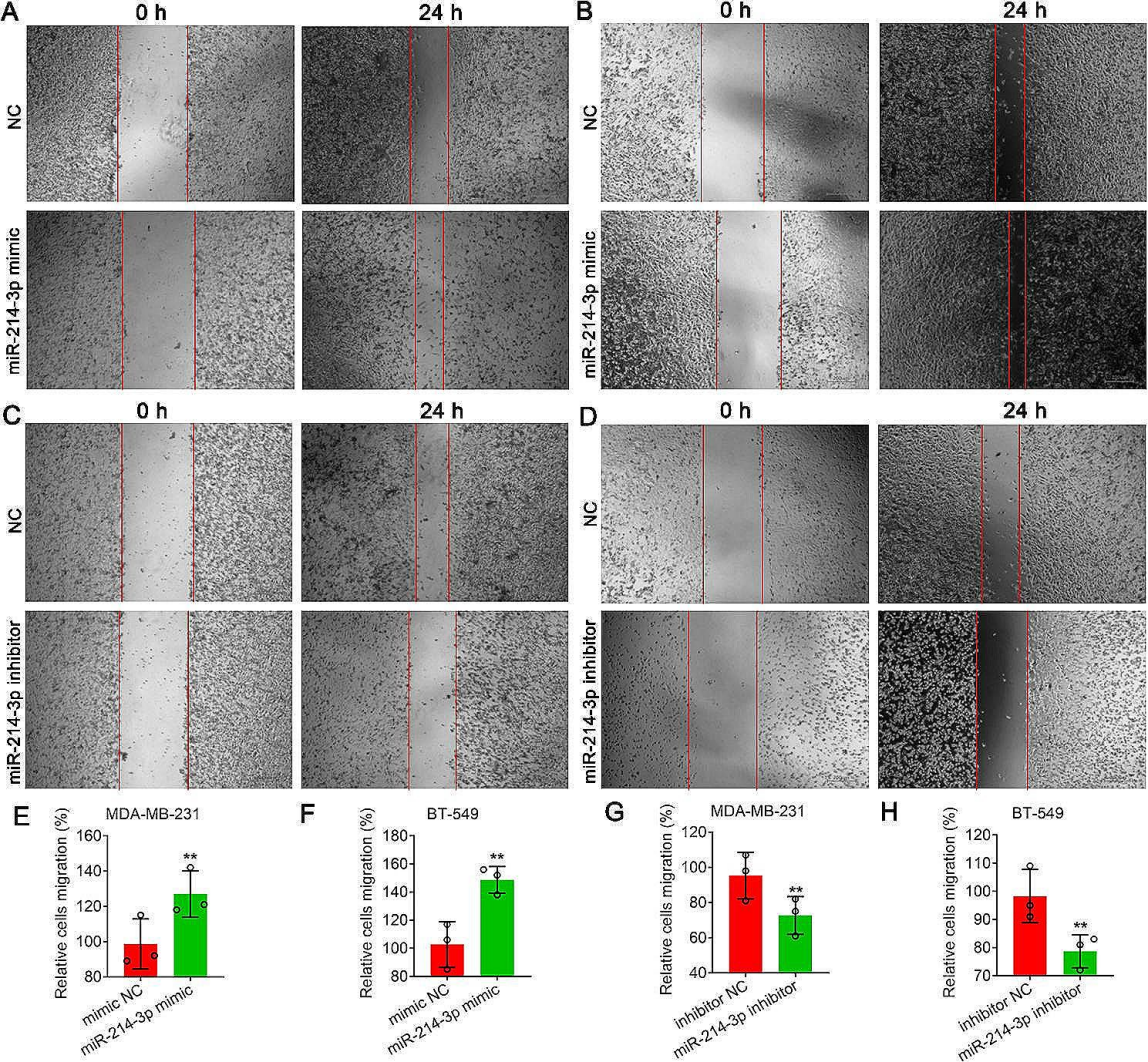
Detection of cell migration via a wound healing assay. (A) and (B) Migration of MDA-MB-231 (**A**) and BT-549 (**B**) cells transiently transfected with miR-214-3p mimics. (**C**) and (**D**) Migration of MDA-MB-231 (**C**) and BT-549 (**D**) cells transiently transfected with miR-214-3p inhibitors. (**E**) and (**H**) Quantitative analysis of cell migration in **A, B, C** and **D**. Bars = 200 μm. The data are presented as the means ± SDs; n ≥ *3*, **P < *0.01*

**Fig. 8:**
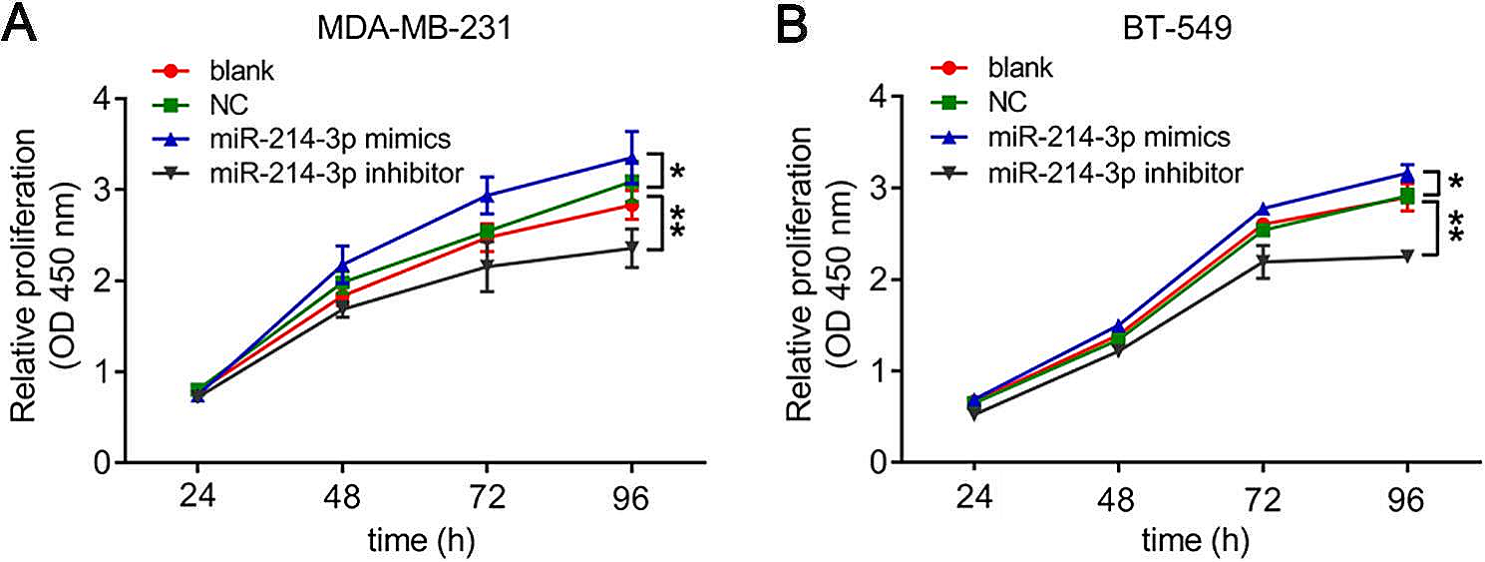
Detection of the relative cell proliferation of MDA-MB-231 (**A**) and BT-549 (**B**) cells transfected with miR-214-3p inhibitors and mimics by CCK8 assay compared to that of the normal controls. The data are presented as the means ± SDs; n ≥ *3*, *P < *0.05*, and **P < *0.01*

The results from the transwell experiment showed that transfection of both MDA-MB-231 and BT-549 cells with the miR-214-3p mimic increased the proportion of invading cells (**P < *0.01*) (Fig. 6A, B, E, F). Conversely, cells transiently transfected with the miR-214-3p inhibitor exhibited significantly lower numbers of invading cells (**P < *0.01*) (Fig. 6C, D, G, H). Furthermore, the wound healing assay revealed that more MDA-MB-231 and BT-549 cells transfected with the miR-214-3p mimic migrated from the wound edge after 24 h, significantly reducing the wound width (**P < *0.01*) (Fig. 7A, B, E, F). However, the opposite results were obtained for MDA-MB-231 and BT-549 cells transfected with the miR-214-3p inhibitor, and the miR-214-3p inhibitor significantly reduced the cell migratory capacity (**P < *0.01*) (Fig. 7C, D, G, H). Consistent with these results, the CCK8 assay showed that compared with the NC, the miR-214-3p mimic significantly increased proliferation after 96 h of transfection in MDA-MB-231 and BT-549 cells, but the miR-214-3p inhibitor significantly decreased proliferation compared with that in the NC group (*P < *0.05*, **P < *0.01*) (Fig. 8A, B).

## Discussion

Many circRNAs have been identified in mammals via the development of high-throughput sequencing techniques and the application of bioinformatics analysis [24]. They have been recognized as key mediators in a wide range of biological processes, and dysfunction of circRNA regulation is responsible for the occurrence and progression of a variety of cancers [25]. In this study, we obtained 78 differentially expressed circRNAs according to microarray analysis in 6 TNBC tissues compared to adjacent noncancerous tissues, which confirmed previous findings that circRNAs are closely associated with various cancers. Further bioinformatics analysis and qRT‒PCR revealed that hsa_circ_0045881 expression was mostly downregulated, which was further confirmed by the subsequent results from the TNBC cell lines MDA-MB-231, MDA-MB-468 and BT-549. Moreover, biological analysis revealed that lentivirus-mediated has_circ_0045881 overexpression in MDA-MB-231 and BT-549 cells significantly reduced their invasion and migration capacity. These results indicated that has_circ_0045881 might play critical roles in TNBC initiation and progression by regulating cell invasion and metastasis. Given the limited functional analysis of circRNAs and their promising role in TNBC treatment [26, 27], we propose that has_circ_0045881 could be a novel diagnostic marker or therapeutic target for TNBC patients in the future.

Altered miR-214-3p is related to the outcomes of various cancers [28]. In addition, osteolytic bone metastasis was reported to significantly increase miR-214-3p expression in BC patients, and abnormally elevated miR-214-3p expression was observed in TNBC tissues [21, 23]. Nasopharyngeal carcinoma patients reportedly exhibit a gradual decrease in miR-214-3p expression after treatment, which is accompanied by increased expression when recurrence or metastasis occurs [29]. Hence, miR-214-3p seems to play key roles in tumour progression in some cancer types, including BC. Moreover, its expression is reportedly decreased in other malignancies, such as hepatocellular carcinoma, lung cancer, and colorectal cancer tissues [19, 30, 31]. Thus, its role might differ according to cancer type. Accumulating data indicate that circRNAs act as modulators of miRNA levels and regulate cellular activity [32, 33]. In this study, we observed that has_circ_0045881 interacted with miR-214-3p in TNBC cells. Moreover, TNBC cells transiently transfected with the miR-214-3p mimic exhibited significant promotion of cell invasion, migration and proliferation, but the miR-214-3p inhibitor exhibited the opposite effects, which was consistent with previous findings [23], indicating that has_circ_0045881 potentially mediates cell invasion and migration in TNBC by sponging miR-214-3p to repress its function.

It should be noticed that the regulatory mechanism of has_circ_0045881/miR-214-3p mainly bases on the studies on human TNBC cancer lines, lack of animal or clinical evidence. Additionally, no studies have ever conclusively demonstrated that has_circ_0045881 are specifically expressed in breast cancer tissues and miR-214-3p role may differ according to cancer types, which increases the difficulty of developing them into biological targets of TNBC. Anyway, more investigation is needed to confirm the potential application of has_circ_0045881/miR-214-3p in clinical treatment for TNBC patients in the future.

## Conclusions

In conclusion, this study revealed that the novel ncRNA has_circ_0045881 is involved in TNBC progression and functions as a sponge for miR-214-3p, thereby modulating the function of miR-214-3p to regulate cell invasion, migration and proliferation. The has_circ_0045881/miR-214-3p axis provides possibilities for the design of TNBC biomarkers or the development of therapeutic strategies.

### Electronic supplementary material

Below is the link to the electronic supplementary material.

Supplementary Material 1

## Data Availability

The datasets used and/or analysed during the current study are available from the corresponding author on reasonable request.

## Abbreviations

TNBC: Triple-negative breast cancer
BC: Breast cancer
GO: Gene ontology
KEGG: Kyoto Encyclopedia of Genes and Genomes analysis
RIP: RNA immunoprecipitation
CCK8: Cell Counting Kit-8
ncRNAs: Noncoding RNAs
circRNAs: Circular RNAs
miRNA: MicroRNA
4EBP-1: 4E binding protein 1
UTRs: Untranslated regions
BMP2: Bone morphogenetic protein 2
PR: Progesterone
ER: Estrogen
HER2: Human epidermal growth factor receptor-2
EMT: Epithelial mesenchymal transition
