# Supplementary material for: The novel circRNA circ_0045881 inhibits cell proliferation and invasion by targeting mir-214-3p in triple-negative breast cancer
Source: BMC Cancer. 2024 Mar 1;24:278. doi: 10.1186/s12885-024-12007-0 (PMC10905830; doi:10.1186/s12885-024-12007-0)
Supplement: Supplementary file 1 — Supplementary Material 1 [file 12885_2024_12007_MOESM1_ESM.docx]

**Supplemental figures**

**
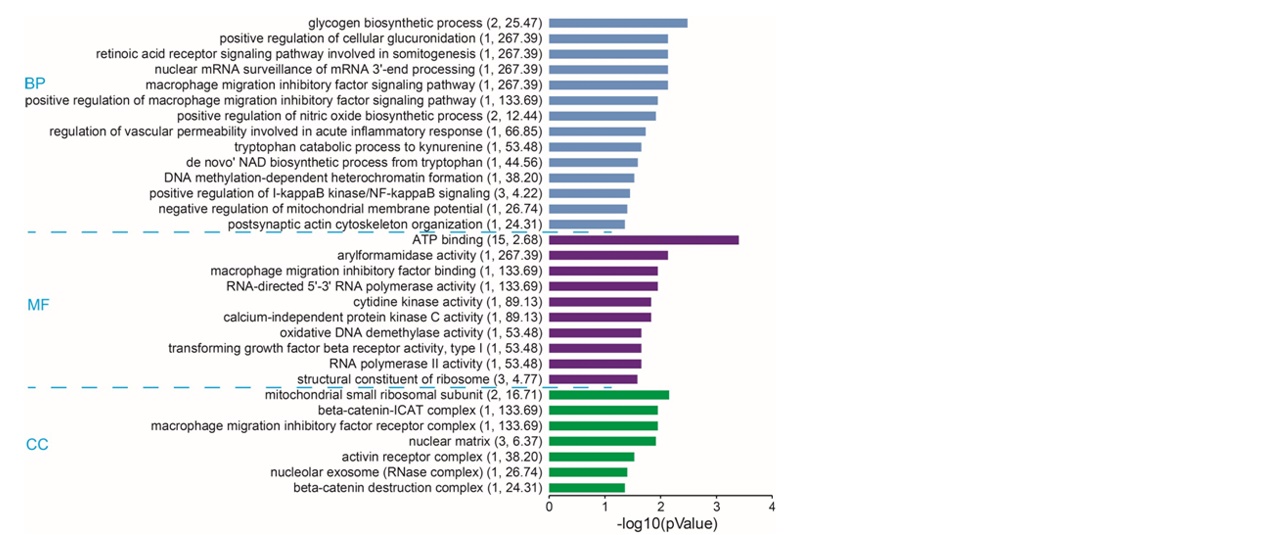
**

**Supplemental Figure 1 The GO analysis of the different expressed circRNAs in TNBC patiens.** BP, biological process, MF, molecular function, and CC, cellular component. X-axis: -log10(P-value). Y-axis: the GO category.

**
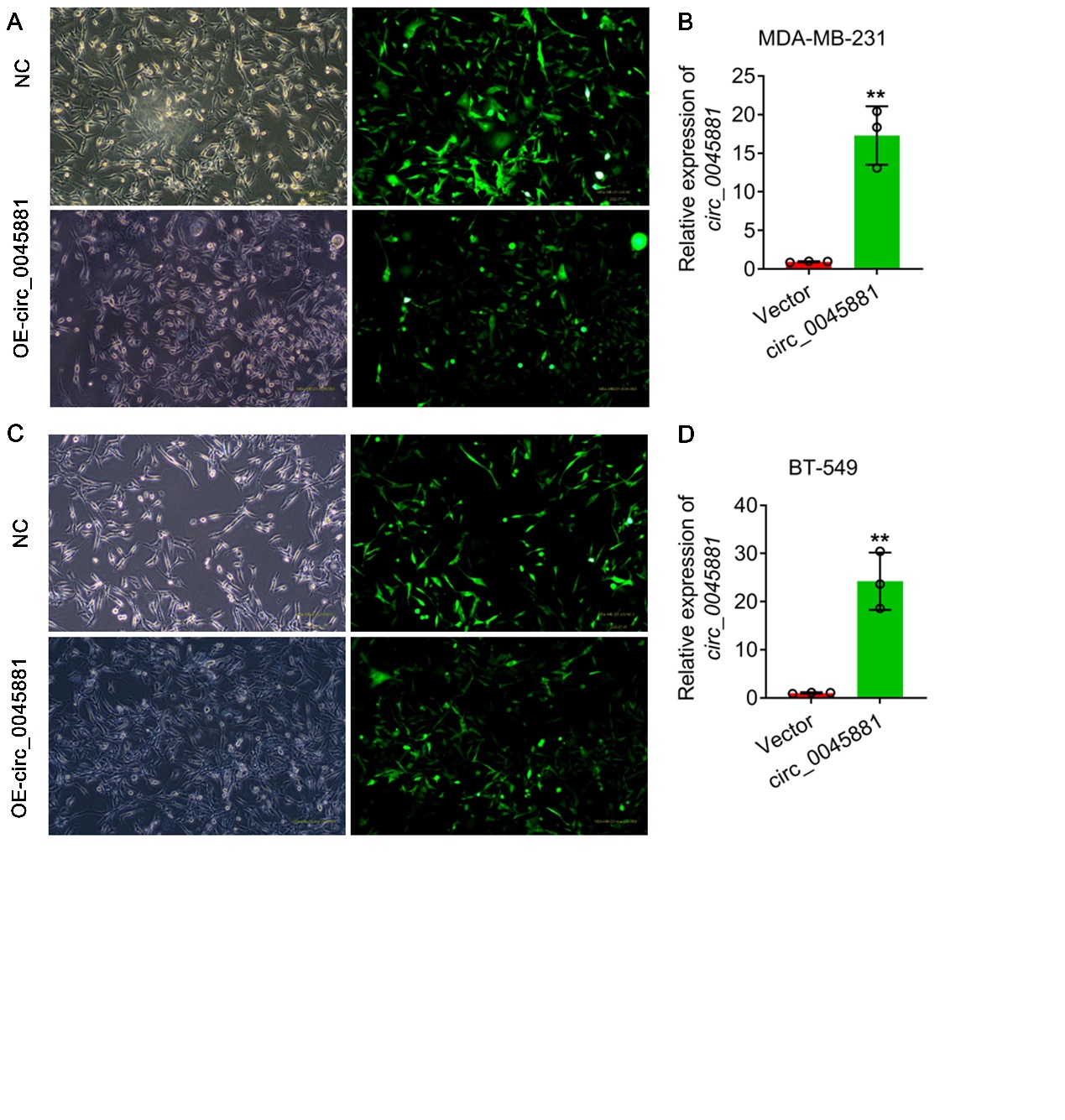
**

**Supplemental Figure 2 The expression of circ_0045881 in MDA-MB-231 and BT-549 cells with lentivirus-delivered circ_0045881 overexpression.** (A) and (C) eGFP signals in MDA-MB-231 (A) and BT-549 cells (C) with lentivirus-delivered OE- circ_0045881. (B) and (D) The relative expression of circ_0045881 in A and C by qRT-PCR. The data are presented as means ± SD, n ≥ *3,* ***P* < *0.01*.


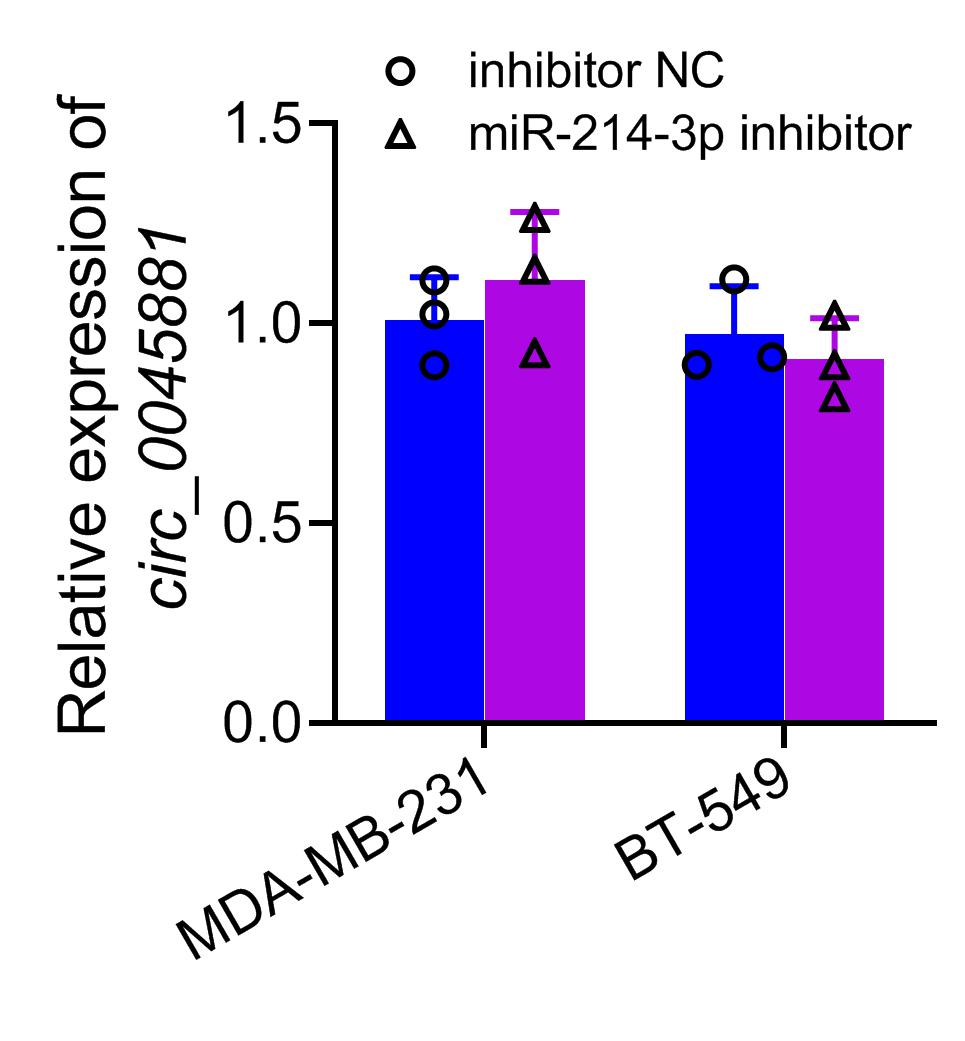


**Supplemental Figure 3 hsa_circ_0045881 expression levels were detected in TNBC cells with miR-214-3p inhibitor.** The data are presented as means ± SD, n ≥ *3,* *P* > *0.05*.


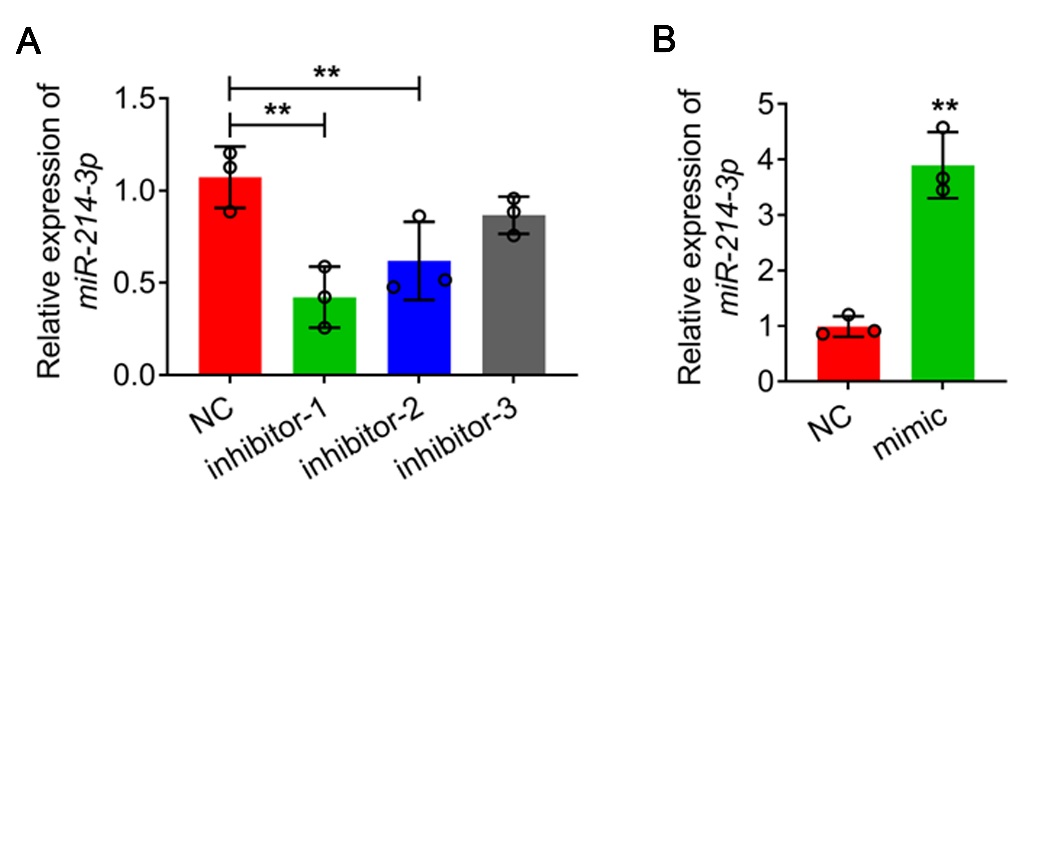


**Supplemental Figure 4 The relative expression of miR-214-3p in MDA-MB-231 cells transiently transfected with miR-214-3p inhibitors (A) and mimics (B).** The data are presented as means ± SD, n ≥ *3,* ***P* < *0.01*.
